# *OsMPH1* regulates plant height and improves grain yield in rice

**DOI:** 10.1371/journal.pone.0180825

**Published:** 2017-07-14

**Authors:** Yongxing Zhang, Chunsheng Yu, Jianzhong Lin, Jun Liu, Bin Liu, Jian Wang, Aobo Huang, Hongyu Li, Tao Zhao

**Affiliations:** 1 Institute of Crop Science, Chinese Academy of Agricultural Sciences, Beijing, China; 2 College of Biology, Hunan Province Key Laboratory of Plant Functional Genomics and Developmental Regulation, Hunan University, Changsha, China; 3 Crop Research Institute, Ningxia Academy of Agriculture and Forestry Sciences, Ningxia, China; Institute of Genetics and Developmental Biology Chinese Academy of Sciences, CHINA

## Abstract

Plant height is a major trait affecting yield potential in rice. Using a large-scale hybrid transcription factor approach, we identified the novel MYB-like transcription factor OsMPH1 (MYB-like gene of Plant Height 1), which is involved in the regulation of plant height in rice. Overexpression of *OsMPH1* leads to increases of plant height and grain yield in rice, while knockdown of *OsMPH1* leads to the opposite phenotypes. Microscopy of longitudinal stem sections indicated that a change in internode cell length resulted in the change in plant height. RNA sequencing (RNA-seq) analysis of transgenic rice lines showed that multiple genes related to cell elongation and cell wall synthesis, which are associated with plant height and yield phenotypes, exhibited an altered expression profile. These results imply that *OsMPH1* might be involved in specific recognition and signal transduction processes related to plant height and yield formation, providing further insights into the mechanisms underlying the regulation of plant height and providing a candidate gene for the efficient improvement of rice yield.

## Introduction

Plant height is an important agronomic trait of rice that directly affects the yield of this crop. The dwarf phenotype is beneficial for rice lodging, but if the plants are too short, it will lead to insufficient growth and ultimately affect the yield potential of rice. Therefore, in an absence of lodging, it is essential to increase plant height to increase yield. The second green revolution and the breeding of super rice are based on appropriate plant heights [1–3]. Therefore, it is of great significance to explore and identify plant height genes and apply them to rice breeding.

Most changes in plant height are related to the length of the internodes, which are altered by changes in the number or length of internode cells. Cell elongation involves turgor-driven expansion through wall component deposition or wall loosening. Cell expansion involves widespread changes in the cell wall architecture in terms of both mass and composition. To undergo expansion, the cell wall must first be softened and relax so that cell wall plasticity is increased and the synthesis of new cell wall material and the amount of protoplasm also increase. Multiple internal and external factors, such as environmental conditions and plant hormones, are involved in the regulation of cell wall-loosening or the deposition of cell wall components. These processes are normally regulated by specific transcription factors, and a number of MYB family genes have been characterized as important regulators in cell wall biosynthesis.

MYB transcription factors are a group of ubiquitous transcription factors that are widely found in plant and animal species. The MYB family is one of the largest families of transcription factors in plants. According to reported statistics, there are 197 *MYB* genes in *Arabidopsis* and 155 in rice [4]. MYB proteins contain a characteristic conserved domain, the MYB DNA-binding domain. Based on the number of MYB domains, the MYB family can be divided into four classes: the 1R-, R2R3-, 3R- and 4R-MYB proteins [5]. *MYB* genes are involved in various processes, such as biological and abiotic stress, development, differentiation, metabolic reactions and defense[6]. At present, functional studies on MYB transcription factors are mainly focused on the regulation of plant responses to environmental stress, although *MYBs* also exhibit important functions in other processes, such as the cell cycle and cell wall biosynthesis. *Arabidopsis* MYB103, MYB85, MYB52, MYB54, MYB69, MYB42, MYB43 and MYB20 are regulators of the biosynthesis of lignin, xylan and cellulose, participating in secondary cell wall thickening [7–10]. MYB46 is a direct regulator of the genes involved in the biosynthesis of all three major components of the secondary wall as well as transcription factors in the biosynthesis pathways [8, 11–13]. *OsMYB46* and *ZmMYB46*, the orthologues of Arabidopsis *MYB46*/*MYB83*, share similar functions and are able to activate the secondary wall biosynthetic program when overexpressed in Arabidopsis [14]. *CEF1/OsMYB103L* and *MYB61* are also involved secondary wall biosynthesis mediated by the GA pathway, which can affect leaf shape, cellulose synthesis and mechanical strength in rice [15, 16].

In this study, we identified a novel rice height-regulating gene that encodes a *MYB* family transcription factor. *OsMPH1* overexpression increased plant height by elongating internode cell length. *OsMPH1* also affected the expression of multiple wall-associated kinase genes, which implies that *OsMPH1* is involved in the regulation of cell development.

## Methods

### Plant materials and growth conditions

The Kita-ake cultivar (*Oryza sativa japonica* cv. Kita-ake) was used as the wild-type. Rice plants were grown at the Experimental Station of the Chinese Academy of Agricultural Sciences in Beijing (39°54′ N, 116°23’ E) under natural conditions from May to October of 2014 to 2016 year. Field experiments were performed with three replicates, and each replicate included 10 individuals for each material. Relevant agronomical traits were recorded at heading and mature stages and analyzed with least significance difference (LSD) software.

### Generation of transgenic rice plants

The construction of *OsMPH1V* and *OsMPH1E* has been described in a previous report [17]. The *OsMPH1* overexpression vector was recombined with the destination vectors pBCV, pBCE [17] and pCAMBIA1301-Bar-FLAG using the Gateway cloning system (Invitrogen). The primers used for this purpose are listed in S1 Table. The constructs were subsequently introduced into *Agrobacterium tumefaciens* strain *EHA105* and then transformed into Kita-ake wild-type plants [18].

### *OsMPH1* bioinformatics analysis

The *OsMPH1* gene locus identifier is *LOC_Os06g45890*. Protein alignment was carried out with ClustalX 2.0. A phylogenetic tree was constructed with MEGA5.1 using the neighbor-joining (NJ) method. The bootstrap values for nodes in the phylogenetic tree came from 1000 replications. The handling gap option was pairwise deletion, and the numbers at the branching points indicate the bootstrap values. The accession numbers and protein sequences are shown in S3 Table.

### Histological analysis

The first internodes in the heading date stage were collected from WT, *OsMPH1V* and *OsMPH1E* plants, then fixed in FAA solution (60% (v/v) ethanol, 5% (v/v) glacial acetic acid and 5% (v/v) formaldehyde) and subjected to vacuum pumping for 40 minutes. Next, the internodes were dehydrated in a series of ethanol solutions (70% (v/v) ethanol, 80% (v/v) ethanol, 85% (v/v) ethanol, 90% (v/v) ethanol, 95% (v/v) ethanol, and anhydrous ethanol) and destained in a series of xylene solutions (3:1 ethanol: xylene, 1:1 ethanol: xylene, 1:3 ethanol: xylene, and pure xylene). The internodes were soaked in each ethanol and xylene solution for two hours and then embedded in paraffin. Tissue sections were cut with a Leica rotary microtome, fixed on a glass slide, and stained with 0.05% Toluidine Blue O (Sigma).

### Subcellular localization

Full-length *OsMPH1* was inserted into the PA7-YFP vector, which had been digested with *Bam*HI and *Sma*I using the In-fusion system (Clontech). OsMPH1-YFP was then transiently expressed in *Arabidopsis* mesophyll cells [19]. The AtAHL-RFP fusion protein was used as a nuclear marker. The resulting fluorescent signal was observed under a confocal microscope (Zeiss LSM700) after 16 h of transformation at room temperature in the dark.

### Transactivation activity assays in yeast

Full-length *OsMPH1* was inserted into the pGBKT7 vector and transformed into the yeast strain AH109. The empty pGBKT7 vector and the BD-4VP16 and BD-4EAR vectors were used as controls. Transformed yeast was grown in SD/-W (-Trp) and SD/-W-H-Ade (-Trp/-His/-Ade) plates for 48 hours before taking photographs. β-galactosidase activity was measured according to the Yeast Protocols Handbook (Clontech) using chlorophenol red-β-D-galactopyranoside as a substrate (CPRG, Roche Biochemical).

### RNA isolation and qRT-PCR analysis

The WT plants and the *OsMPH1V* and *OsMPH1E* transgenic lines were cultivated under continuous light at 28°C for 4 weeks in plant growth chambers. RNA extraction and quantitative reverse transcription PCR (qRT-PCR) analysis were performed as described previously [20].

### Immunoblots

Immunoblot analysis was performed using one-week-old seedlings as described previously [20].

### RNA-seq and data analysis

The WT plants and the *OsMPH1V*-3and *OsMPH1E-22* were grown under continuous light at 28°C till four leaves stage and total RNA was extracted using the Trizol reagent (Invitrogen). The sequencing library was constructed following the manufacturer’s instructions (Illumina Inc.). Paired-end sequencing libraries with an insert size of approximately 200 bp were sequenced on an Illumina HiSeq 2000 sequencer at ANOROAD Company in Beijing. RNA-seq clean reads of three biological replicates were mapped to the *O*. *sativa ssp*. *japonica* reference genome after removing adaptor and low quality nucleotides by TopHat. The expression value was calculated in FPKM (fragments per kilobase of exon model per million mapped fragments) and the differentially expressed genes were further analyzed by Cuffdiff (q < 0.05). Differentially expressed genes were defined as those with fold changes ≥ 2, or ≤ 2/3. RNA-seq data have been deposited in the ArrayExpress database at EMBL-EBI (https://www.ebi.ac.uk/arrayexpress/experiments/E-MTAB-5759) under accession number E-MTAB-5759.

## Results

### *OsMPH1 (MYB-like gene of plant height 1)* is involved in the regulation of plant height

Previously, we described the approach of using hybrid transcription factors (HTFs) to investigate the roles of different TFs in plant growth and development [17]. By surveying the phenotypes of over 50,000 transgenic lines, covering 1,500 rice transcription factors fused with the transcription activation module 4VP16 or the repression module 4EAR, we identified a pair of HTFs that exhibited opposite plant height phenotypes, referred to as OsMPH1-4VP16 (OsMPH1V) and OsMPH1-4EAR (OsMPH1E). In total, we obtained 34 *OsMPH1V* transgenic events (designated as *OsMPH1V*s) and 25 *OsMPH1E* transgenic events (designated as *OsMPH1E*s), among which 19 *OsMPH1Vs* and 15 *OsMPH1Es* showed a dwarf or taller phenotype, respectively. We verified the transgenic plants via quantitative reverse transcription PCR (qRT-PCR) or immunoblot analyses and selected two *OsMPH1Vs* and two *OsMPH1Es* for further analyses (S1A and S1B Fig).

**Fig 1:**
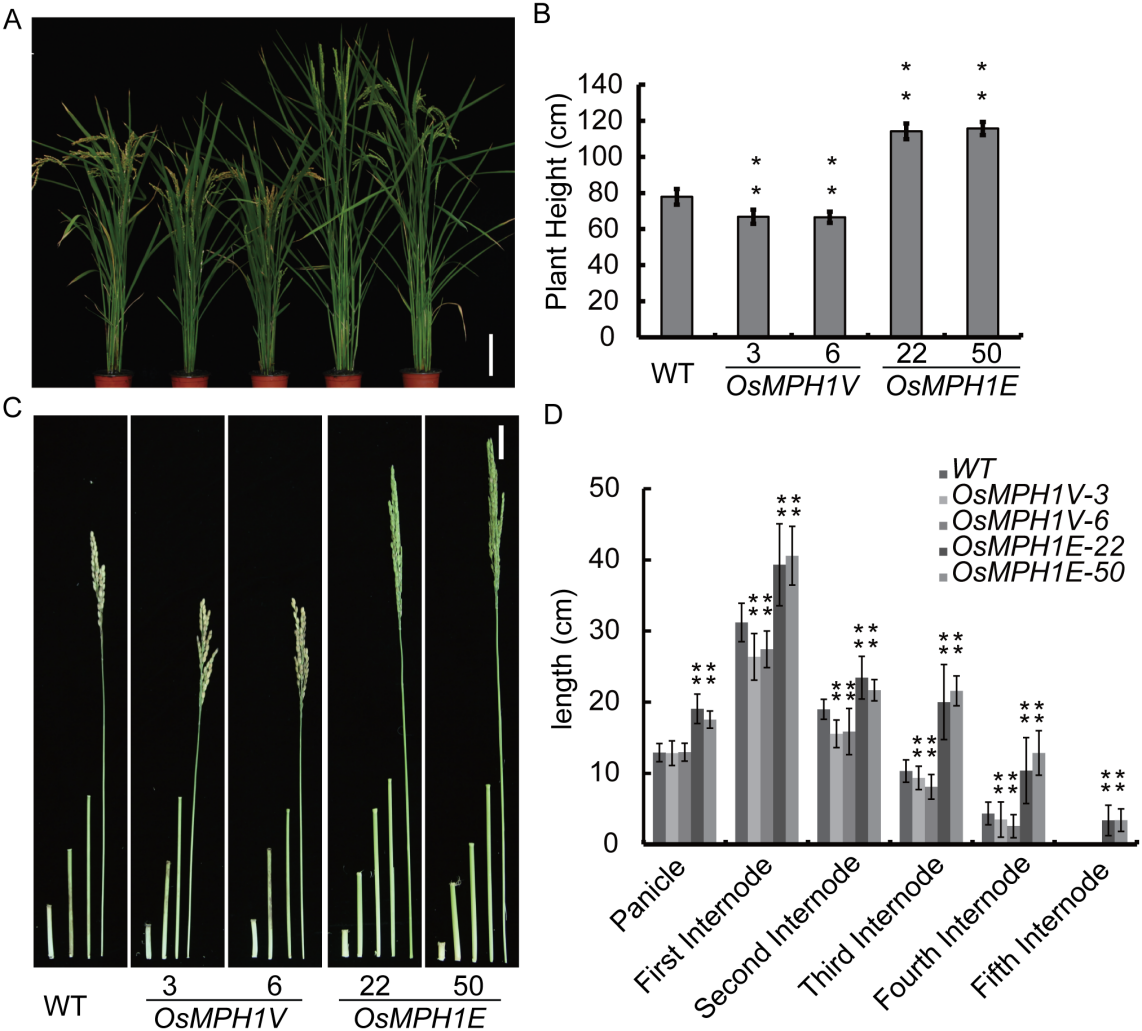
Phenotypic analysis of *OsMPH1V-* and *OsMPH1E*-overexpressing plants. **A** Gross morphology of WT, *OsMPH1V* and *OsMPH1E*. Bars = 20 cm. **B** Comparison of plant height between WT, *OsMPH1V* and *OsMPH1E* transgenic rice. Data are shown as the means ± s.d. (Student’s *t* tests, **P < 0.01, n = 60). **C** Internode morphology of WT, *OsMPH1V* and *OsMPH1E*. Bars = 5 cm. **D** Schematic representation and comparison of the various elongation patterns of internodes in WT, *OsMPH1V* and *OsMPH1E* transgenic rice. Data are shown as mean as the means ± s.d. (Student’s *t* tests, **P < 0.01, n = 10).

We first measured plant height. In comparison with the wild-type, the plant height of *OsMPH1Vs-3* and *OsMPH1Vs-6* was decreased by 22.3% and 22.5%, while that of *OsMPH1E*-*22* and *OsMPH1E*-*50* was increased by 41.3% and 44.8%, respectively (Fig 1A and 1B). To confirm the culms phenotype, we measured internode length in the late stage of rice growth. The statistical results showed that the internode length of all *OsMPH1E-22* and *OsMPH1E-50* plants was increased dramatically in comparison with the wild-type; in contrast, the internode length of all *OsMPH1Vs-3* and *OsMPH1Vs-6* plants was markedly decreased(Fig 1C and 1D).

**Fig 2:**
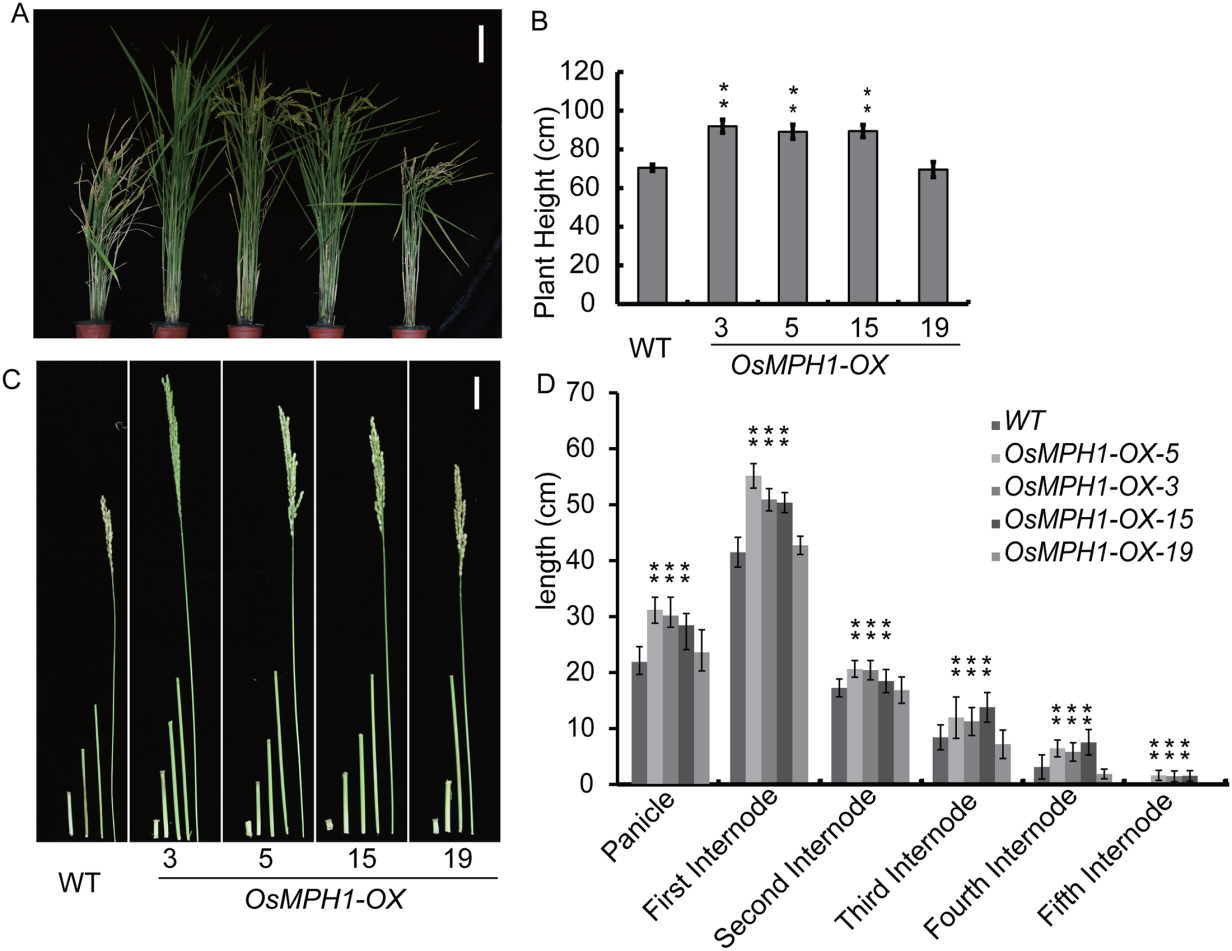
Phenotypic analysis of *OsMPH1-*overexpressing plants. **A** Gross morphology of WT and *OsMPH1-OX*. Bars = 20 cm.**B** Comparison of plant height between WT and *OsMPH1* transgenic rice. Data are shown as the means ± s.d. (Student’s *t* tests, **P < 0.01, n = 60). **C** Internode morphology of WT and *OsMPH1-OX*. Bars = 5 cm. **D** Schematic representation and comparison of the various elongation patterns of internodes in WT and *OsMPH1-OX* transgenic rice. Data are shown as the means ± s.d. (Student’s *t* tests, **P < 0.01, n = 10).

To avoid the artifactual phenotype caused by an additional 4VP16 activation domain or 4EAR suppression domain, we constructed an *OsMPH1* overexpression vector (*OsMPH1-OX)* and obtained 47 independent *OsMPH1-OX* events. Plant height was increased by most of the transgenic events (Fig 2A and 2B). We validated the transgenic plants through qRT-PCR and immunoblot probing with an anti-Flag antibody and selected four representative events for further analysis (S1C and S1D Fig). The statistical results showed that there was correlation between *OsMPH1-OX* plant height and the protein expression level. The greater the amount of the *OsMPH1* protein, the greater the height of *OsMPH1-OX* plants, suggesting that OsMPH1 is involved in the regulation of plant height. Next, we observed the internode length of *OsMPH1-OX* plants. The *OsMPH1-OX* plants displayed a similar phenotype to the *OsMPH1E* plants, with all plants exhibiting a dramatic increase in internode length (Fig 2C and 2D).

We also obtained *OsMPH1-RNAi* transgenic rice and verified that mRNA levels were significantly reduced in these plants (S2A and S2B Fig). In comparison with wild-type plants, the *OsMPH1-RNAi* plants exhibited decreased plant height phenotypes (S2C Fig).

### *OsMPH1* affects internode cell length

**Fig 3:**
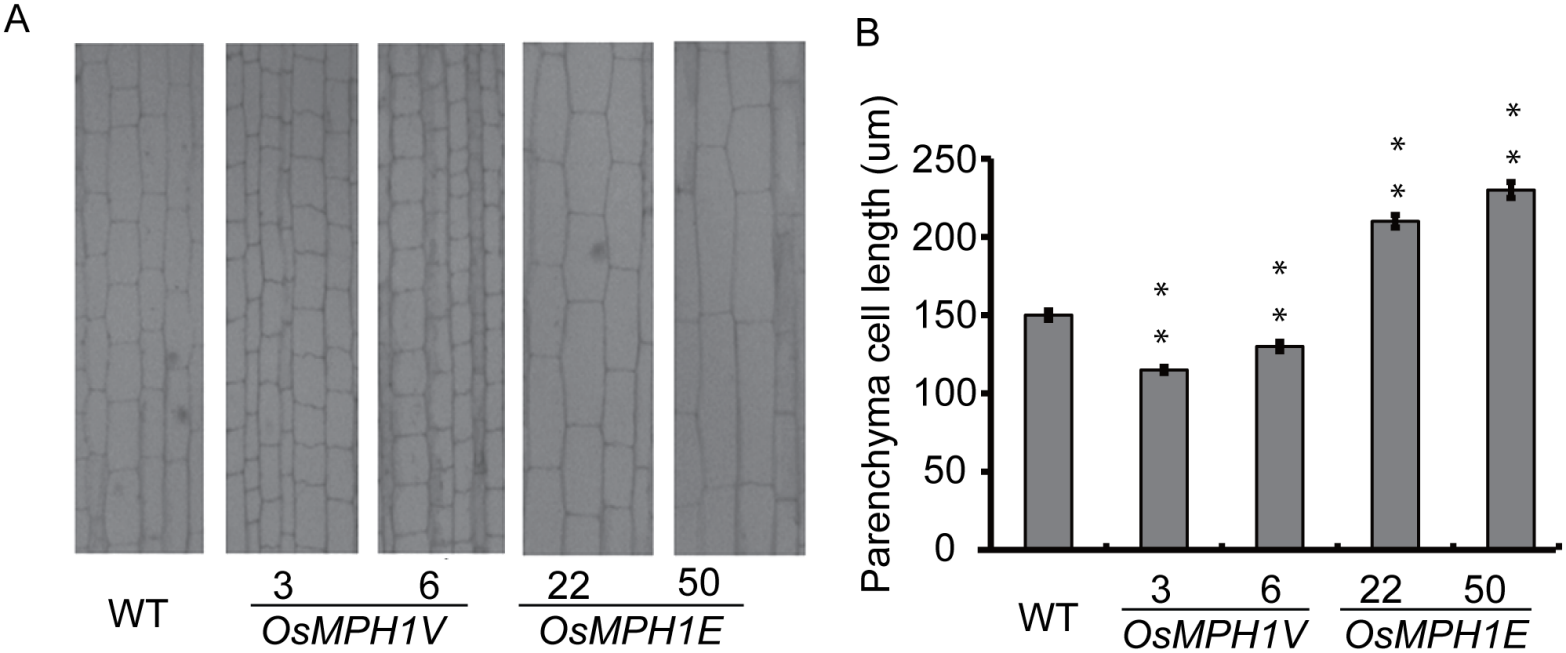
Histological observations of *OsMPH1V* and *OsMPH1E* internodes. **A** Longitudinal section of the first internode from *OsMPH1V* and *OsMPH1E* in the heading stage. Bars = 100 μm. **B** Statistical analysis of internode cell length in *OsMPH1V* and *OsMPH1E*. Data are shown as the means ± s.d. (Student’s t tests, **P < 0.01, n = 10).

The variation in plant height could be caused by a change of internode cell length. To verify this hypothesis, longitudinal anatomical sections of the internodes were analyzed. Longitudinal sections from rice plants were photographed at high magnification under a microscope, and software was used to calculate cell length. The results showed that the longitudinal length of *OsMPH1V* and *OsMPH1E* internode cells was dramatically altered compared with the WT controls. The longitudinal cell phenotype and length statistics are shown in Fig 3. The length of *OsMPH1V* parenchyma cells was significantly decreased, while the length of *OsMPH1E* parenchyma cells was significantly increased, whereas there was no significant change in the number of cells. These results indicated that *OsMPH1* could regulate plant height by altering the length of internode cells.

### *OsMPH1* improves quantitative yield components in rice

**Fig 4:**
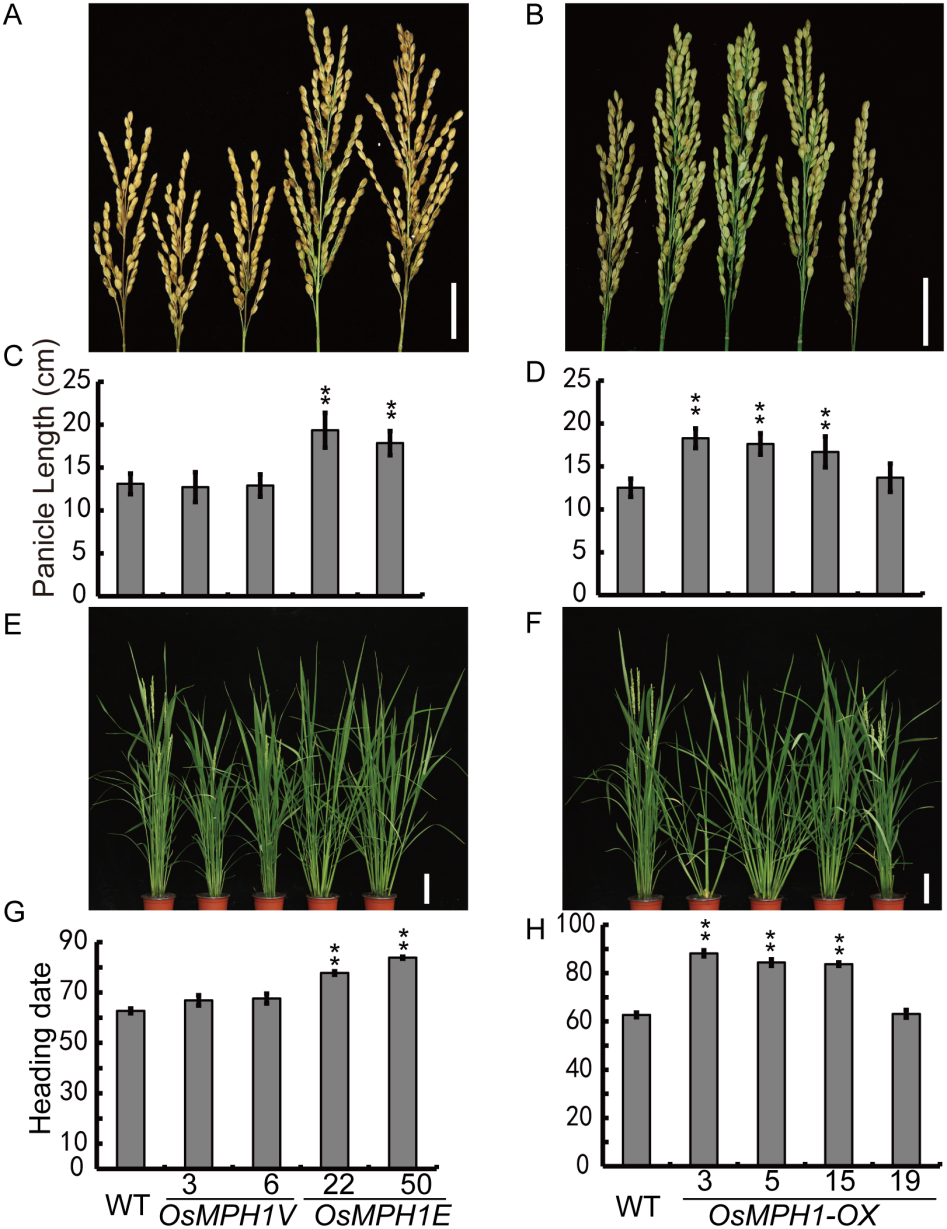
Analysis of plant height and heading dates in *OsMPH1V*, *OsMPH1E* and *OsMPH1-OX* plants. **A,B** Panicle morphology of WT, *OsMPH1V*, *OsMPH1E* and *OsMPH1-OX* transgenic rice **C,D** Comparison of panicle length between WT, *OsMPH1V*, *OsMPH1E* and *OsMPH1-OX* transgenic rice. Data are shown as the means ± s.d. (Student’s *t* tests, **P < 0.01, n = 60). **E,F** Morphology of WT, *OsMPH1V*, *OsMPH1E* and *OsMPH1-OX* transgenic rice on the heading date **G,H** Comparison of heading dates between WT, *OsMPH1V*, *OsMPH1E* and *OsMPH1-OX* transgenic rice. Data are shown as the means ± s.d. (Student’s *t* tests, **P < 0.01, n = 60).

In addition to the greater plant height, we also observed that both *OsMPH1Es and OsMPH1-OXs* showed large-panicle and late-flowering phenotypes under natural conditions (Fig 4).

**Fig 5:**
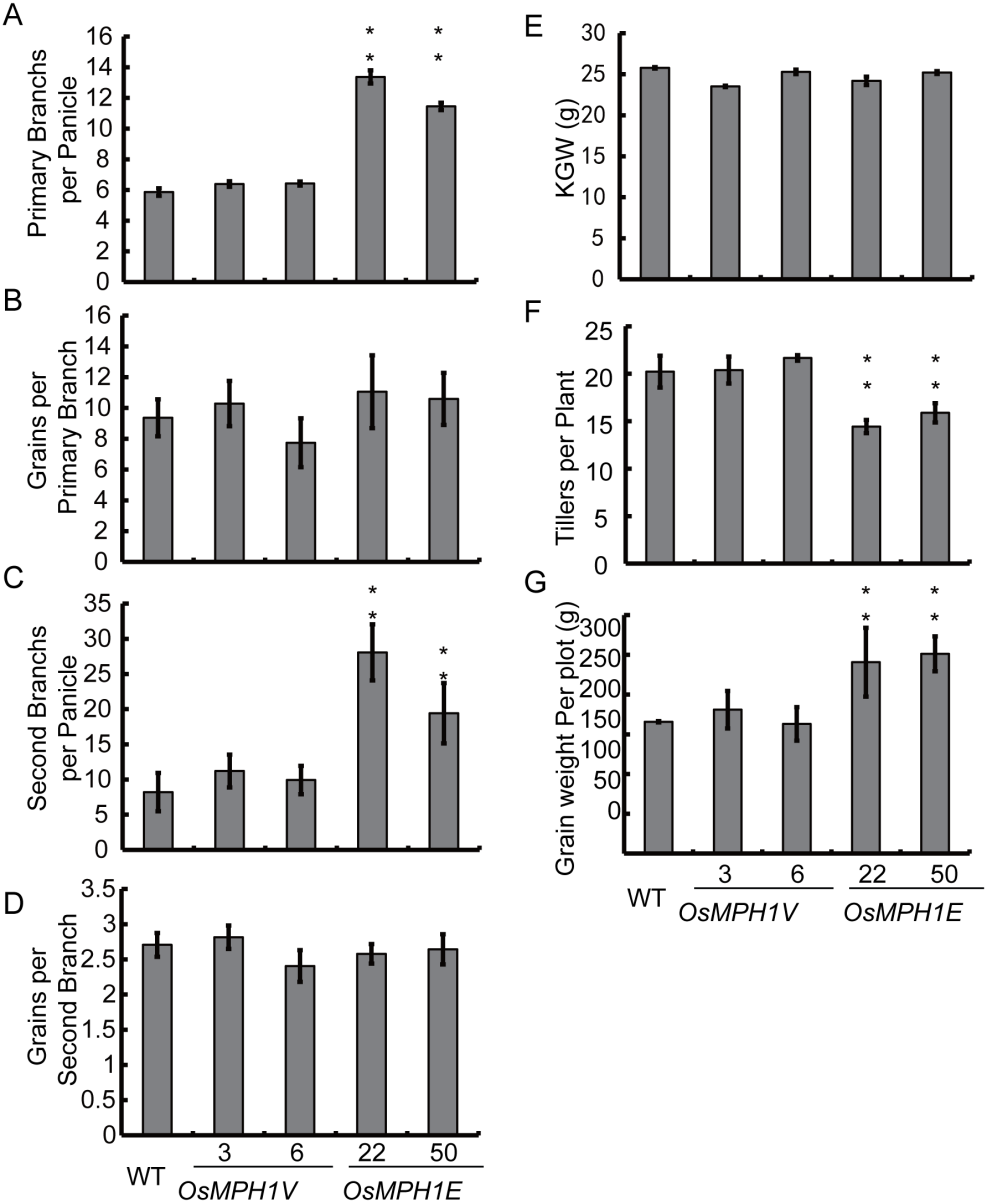
Analysis of yield traits in *OsMPH1V* and *OsMPH1E* plants. **A-G** Comparison of the number of primary branches per panicle, grains per primary branch, secondary branches per panicle, grains per secondary branch, grains per plant, tillers per plant, grain weight per plot and plant height between WT, *OsMPH1V* and *OsMPH1E* transgenic rice. Data are shown as the means ± s.d. (Student’s *t* tests, *P < 0.05, **P < 0.01, n = 60).

To analyze the large-panicle phenotype, we compare the branching pattern of *OsMPH1Vs* and *OsMPH1Es* with the wild-type in more detail. The statistical results indicated that primary and secondary branches were significantly more abundant in *OsMPH1Es* than in the wild-type, while *OsMPH1Vs* exhibited no difference from the wild-type (Fig 5A and 5C). We next counted the grains produced on primary and secondary branches (Fig 5B and 5D). The results showed that the *OsMPH1Vs* and *OsMPH1Es* presented no difference from the wild-type. We also examined other components of the rice yield trait, such as tiller number and 1000-grain weight. Statistical analysis revealed that the *OsMPH1Es* exhibited fewer tillers than the *OsMPH1Vs* and the wild-type, while the 1000-grain weight showed no difference between *OsMPH1V*, *OsMPH1E* and wild-type plants ((Fig 5E and 5F). We further measured the actual grain yield at the plot level (10 plants per plot); the results showed that the grain yields of *OsMPH1Es* were increased by 45% to 50% in comparison with wild-type plants (Fig 5G). The increased number of primary and secondary branches and extended growth period appeared to explain why *OsMPH1Es* exhibit a higher grain yield.

We also checked the grain weight per plot, KGW, tiller number and grains per panicle of *OsMPH1-OXs* and *OsMPH1-RNAi*, which share the similar results to *OsMPH1Es* and *OsMPH1Vs* (S3 Fig).

### Rice *OsMPH1* encodes a MYB family transcription factor

**Fig 6:**
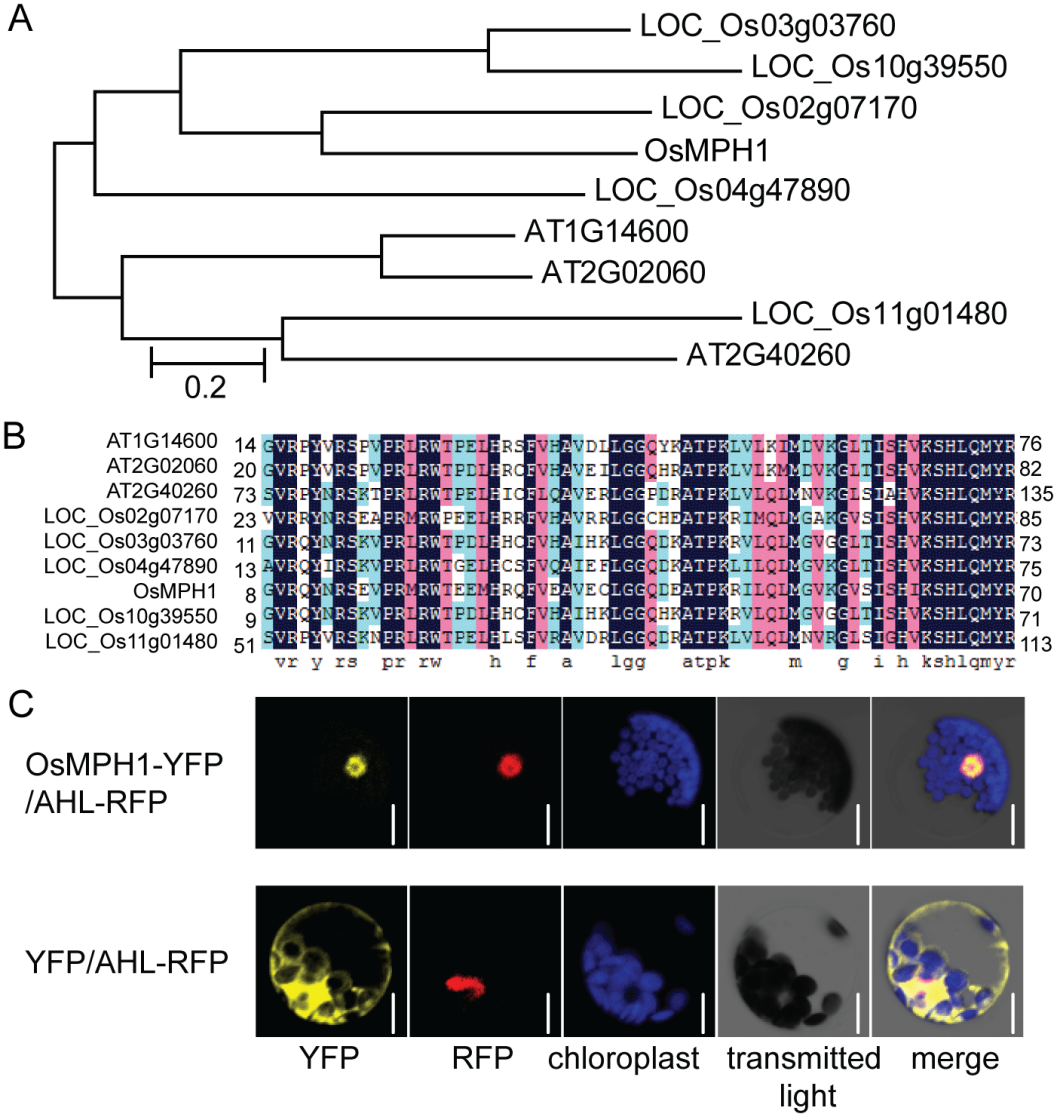
*OsMPH1* encodes a MYB family protein. **A** Phylogenetic tree analysis of the OsMPH1 protein and its rice and *Arabidopsis* homologues. **B** Amino acid alignment of the OsMPH1 protein and its *Arabidopsis* and rice homologues. Conserved amino acids are highlighted in black. **C** Subcellular localization of OsMPH1. The OsMPH1-YFP fusion proteins were located at the plasma membrane and in the nucleus. YFP was used as a control. The AHL-RFP fusion protein was used as a nuclear marker. Bars = 10 μm.

*OsMPH1* refers to *LOC_Os06g45890*, which encodes a 256-amino acid protein with a predicted molecular mass of 28.5 kD. Phylogenetic tree analysis showed that OsMPH1 clustered with homologues in rice (LOC_Os02g07170, LOC_Os03g03760, LOC_Os04g47890, LOC_Os10g39550 and LOC_Os11g01480) and *Arabidopsis* (AT1G14600, AT2G02060 and AT2G40260) (Fig 6A). Protein structural analysis revealed that *OsMPH1* contains a typical R1 Myb_DNA-binding motif from amino acid residues 19 to 70 at the N-terminus. Amino acid alignment showed that OsMPH1 shares over 60% identity with *Arabidopsis* and rice homologues in the Myb_DNA-binding motif, while sharing less than 25% identity in other regions (Fig 6B).

The MYB family transcription factors have been reported to localize to the nucleus. To examine whether OsMPH1 localizes to the nucleus, OsMPH1 was fused with YFP at C-terminus and transiently expressed in the *Arabidopsis* mesophyll protoplasts. The results showed that OsMPH1-YFP was exclusively located in the nucleus, co-localizing with the nuclear marker protein AHL-RFP. In contrast, the control YFP protein was only detectable in the intracellular region (Fig 6C). Together, these data suggest that OsMPH1 is a nuclear-localized MYB family transcription factor.

### Rice *OsMPH1* expression pattern

**Fig 7:**
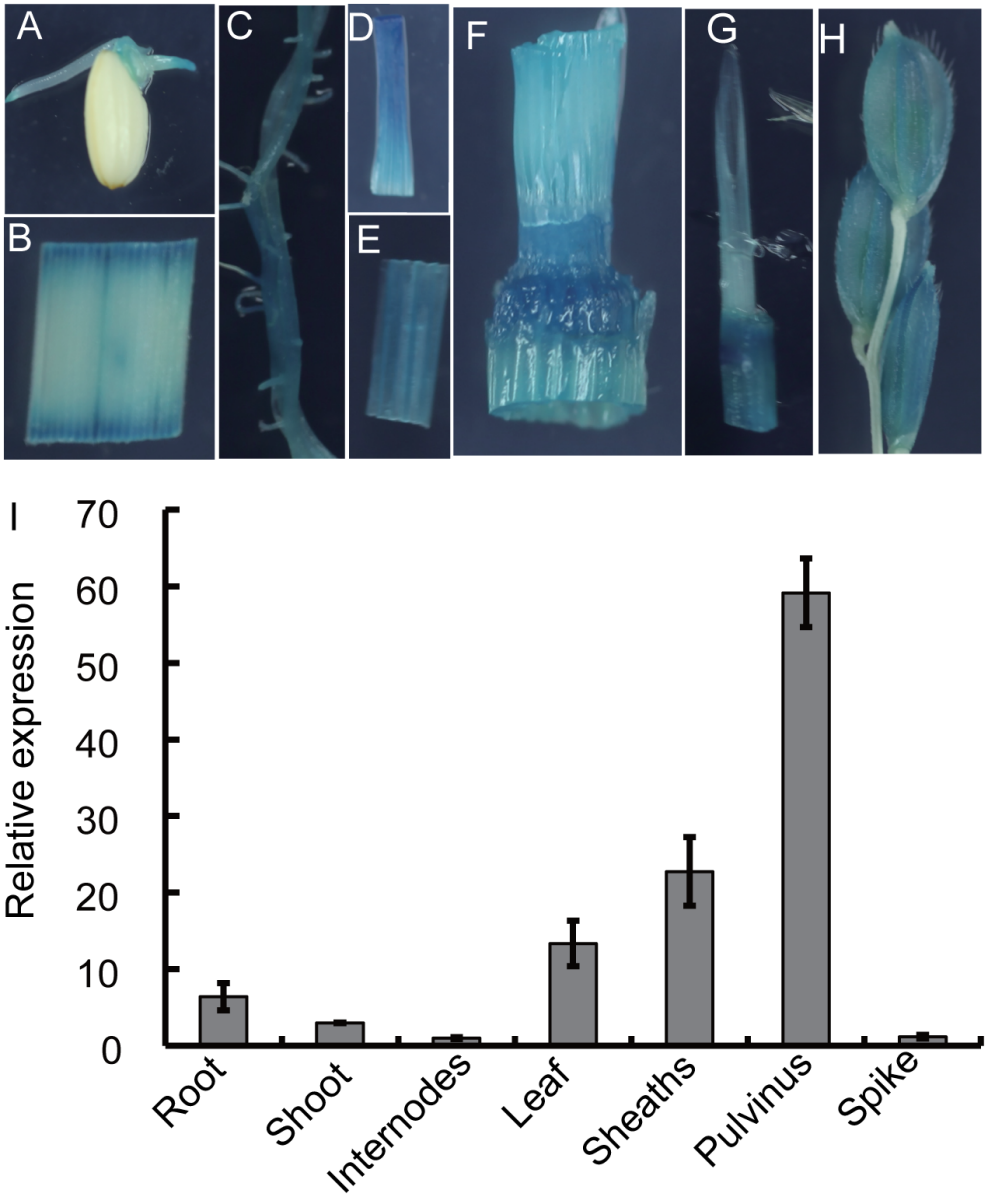
Analysis of the *OsMPH1* expression pattern in rice. **A-H** *OsMPH1* expression revealed by GUS staining in *OsMPH1* promoter-GUS transgenic plants. **A** Germinated seed (2 day); **B** leaf, **C** root; **D** sheath; **E** shoot; **F** stem node; **G** pulvinus; **H** spike. **I** Expression pattern analysis of *OsMPH1* in various vegetative organs via quantitative RT-PCR. Data are shown as the means ± s.d. (n = 3).

To investigate the tissue-specific expression pattern of *OsMPH1*, the GUS reporter system was used to monitor its expression throughout the life cycle (from seeding to mature caryopses). As shown in Fig 7A, histochemical staining indicated that OsMPH1 was expressed in almost all the examined tissues, including the plumule of germinating seeds, coleoptile, leaves, stem nodes, internodes, sheaths, pulvinus, spikes and roots. Among these tissues, OsMPH1 exhibited extremely high expression in the pulvinus and stem nodes.

We also monitored the *OsMPH1* expression pattern using qRT-PCR assays. Similar to the GUS staining results, *OsMPH1* was detectable in all tissues. Notably, our results showed an extremely high mRNA expression level of *OsMPH1* in the leaves and sheaths and especially in the pulvinus, whereas *OsMPH1* was weakly expressed in the internodes and spikes (Fig 7B).

### Rice *OsMPH1* exhibits no self-activation activity

**Fig 8:**
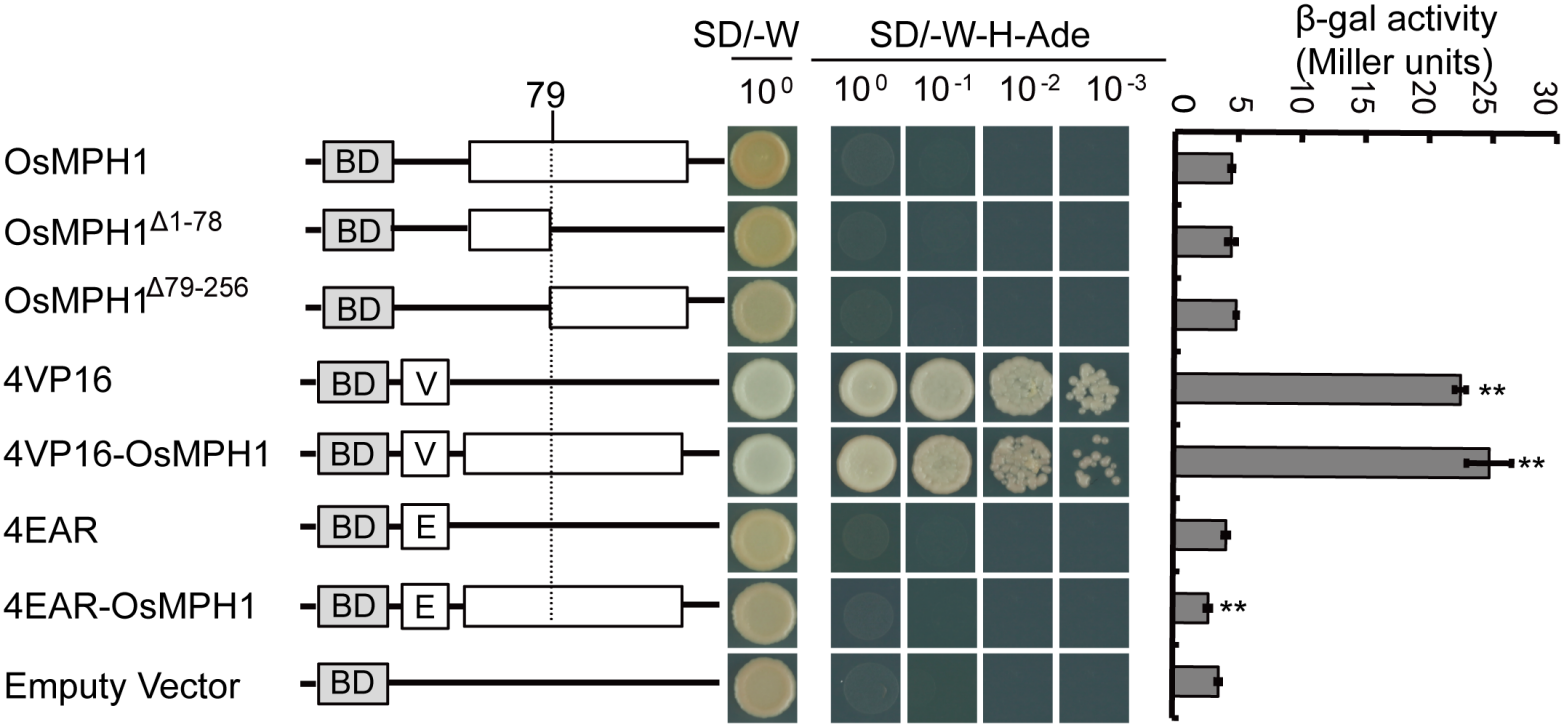
Transcription activation analysis of OsMPH1 in the yeast GAL4 system. Each transformed yeast strain was dropped onto SD/-W and SD/-W-H-Ade plates and allowed to grow for 48 hours before taking photographs. β-galactosidase activity was quantified through a liquid culture assay using CPRG as the substrate. Data are shown as the means ± s.d. (Student’s t tests, **P < 0.01, n = 3).

To determine the transcriptional activity of OsMPH1, constructs of *OsMPH1*, *MPH1V* and *OsMPH1E* with the pGBKT7 vector were generated and used to transform the AH109 yeast cell line. The empty BD vector, 4EAR vector and 4VP16 vector were used as controls. As shown in Fig 8, all the yeast transformants grew well on (-Trp) SD medium. When they were transferred to (-Trp/-His/-Ade) SD medium, only *OsMPH1Vs* and *4VP16s* grew well. We also quantified the β-galactosidase activity of all the transformants using CPRG as a substrate. In comparison with the BD empty vector control, OsMPH1 and its truncated version possessed very low transcriptional activity, while when OsMPH1 was fused with the 4VP16 activation domain, OsMPH1 transcriptional activity increased dramatically. In contrast, when OsMPH1 was fused with the 4EAR suppression domain, OsMPH1 transcriptional activity decreased slightly. These results indicated that OsMPH1V functions as a transcriptional activator, while OsMPH1E functions as a suppressor. OsMPH1 possessed very weak transcriptional activity, and the recruitment of a partner may be necessary for its functional implementation.

### mRNA expression pattern analysis of transgenic plants

**Fig 9:**
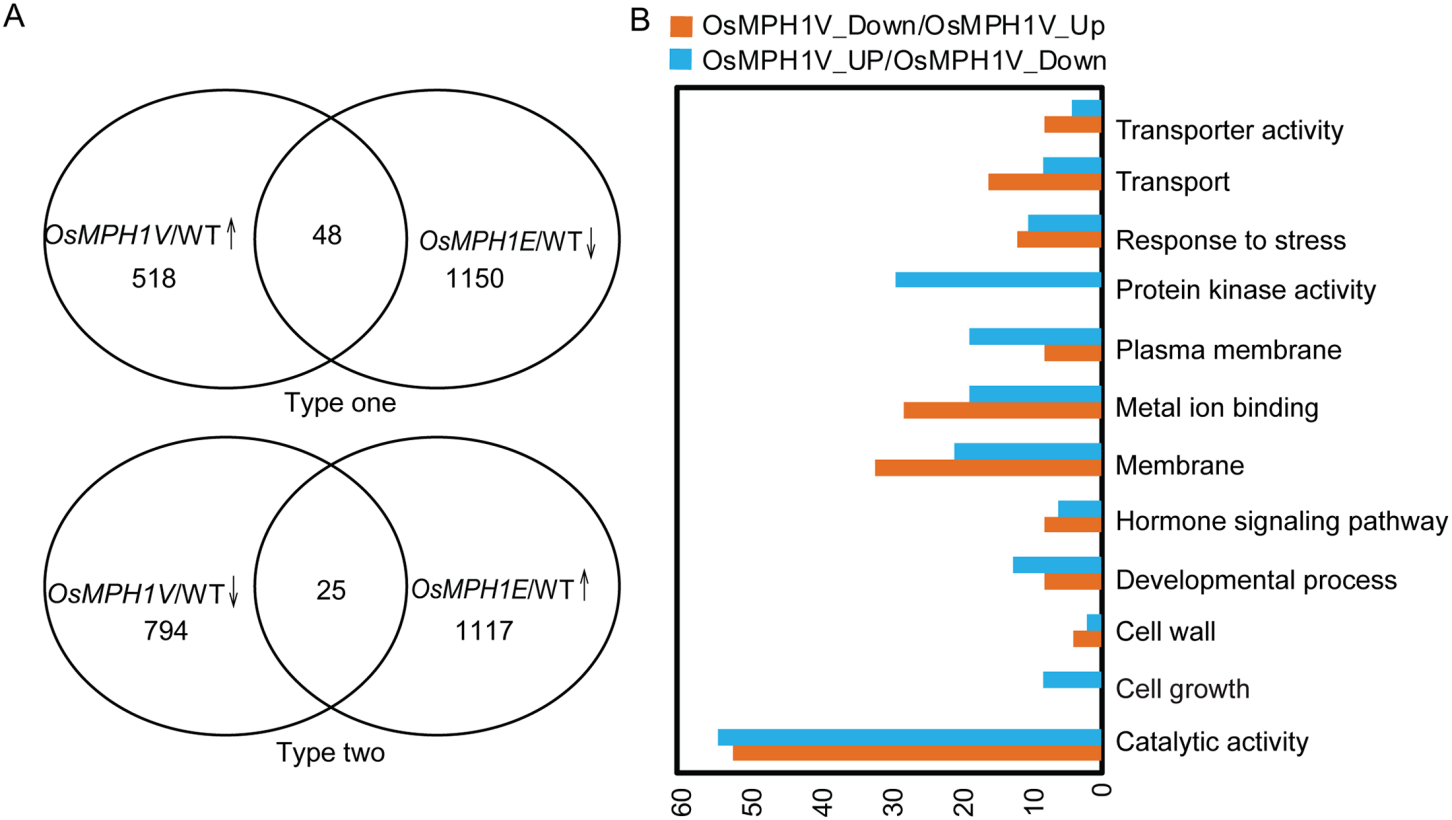
Identification of *OsMPH1*-regulated genes through RNA-seq analysis. **A** Type one Venn diagram showing the overlapping profile of up-regulated genes in the *OsMPH1V* transgenic line and down-regulated genes in the *OsMPH1E* line in comparison with the WT, as determined via RNA-seq; Type two Venn diagram showing the overlapping profile of down-regulated genes in the *OsMPH1V* transgenic line and up-regulated genes in the *OsMPH1E* line in comparison with the WT, as determined via RNA-seq. **B**, Type one and Type two gene function categories.

Our data indicated that a change in internode cell length led to the observed plant height phenotype, which suggests that *OsMPH1* is involved cell length regulation. To further elucidate the regulation of *OsMPH1*, we compared mRNA expression in WT and *OsMPH1V* and *OsMPH1E* transgenic plants using RNA-seq assays (S4 Fig). We identified 73 genes that exhibited opposite expression pattern in *OsMPH1Vs* versus *OsMPH1Es*, which could be divided into two types (Fig 9; S2 Table). The Type-one genes included 48 genes whose expression levels were increased in *OsMPH1Vs* compared with the wild-type but were decreased sharply in *OsMPH1Es*. The Type-two genes included 25 genes whose expression levels were decreased in *OsMPH1Vs*, but dramatically increased in *OsMPH1Es*. Among the Type-one genes, there were 9 receptor-like protein kinases (RLKs), which could be classified into 4 types, including 4 wall-associated (WAK) RLKs (*LOC*_*Os02g42150*, *LOC*_Os04g30010, *LOC*_*Os09g29560* and *LOC*_*Os04g5103*,), 3 leucine-rich repeat (LRR) RLKs, 1 stress-antifung RLK and 1 cysteine-rich RLK. All of these RLKs were predicted to possess a transmembrane region and to localize to the plasma membrane (S2 Table). WAKs are involved in determining the cell wall composition as well as phosphorylation or transcription processes and are required for cell expansion during plant development [21]. We also identified a cinnamyl alcohol dehydrogenase (CAD) (*LOC*_*Os06g22919)* and a xyloglucan endotransglucosylase /hydrolase *(LOC*_*Os04g1592)*, which are key enzymes involved in lignin biosynthesis and the process of plant cell wall remodeling. These genes might provide clues to explain the phenotypic variation of plant height.

In addition, we identified several genes involved in the indole biosynthetic pathway, including two genes (*LOC*_*Os04g08828* and *LOC*_*Os06g02019*) encoding cytochrome P450 monooxygenases, which could convert Trp to indole-3-acetaldoxime (IAOx), a precursor of IAA and indole glucosinolates; one gene (*LOC*_*Os04g09604*) encoding an indole glucosinolate O-methyltransferase; and one gene (*LOC*_*Os05g37470*) encoding an auxin transporter, all of which are involved in cell expansion.

## Discussion

Plant height is closely related to biomass production, which makes it an important morphological trait that affects yield performance. Rice is an economically important crop, in which the ratio of economic output to biological yield is 1:1. Therefore plant height and yield are closely related, and within a certain range, when plant height is increased, the yield is also increased. A moderate plant height is an important basis for rice breeding. In the present study, we identified a new rice *MYB* gene, *OsMPH1*, which might function in plant height regulation. *OsMPH1* is highly expressed in the rice pulvinus and nodes, and the encoded protein is exclusively localized in the nucleus. *OsMPH1* overexpression leads to an increase of plant height, caused by longitudinal elongation of internode cells. Our result also indicated that *OsMPH1Es* plants shared the similar phenotype with *OsMPH1* overexpression plants, while *OsMPH1V* and *OsMPH1-RNAi* plants exhibited the opposite phenotype. Combining with the transcription activation analysis result that OsMPH1V functions as a transcriptional activator and OsMPH1E functions as a suppressor, OsMPH1 might work as a transcriptional suppressor in vivo. Considering that OsMPH1 bears almost no transcriptional activity, recruitment of a partner may be necessary for OsMPH1 to implement the activator function of the plant height.Plant cell elongation growth is regulated by a variety of internal and external factors, and the regulation of endogenous hormones in plants plays an important role in this process. For example, auxin (IAA), gibberellin (GA), brassinolide (BR) and ethylene (ETH) can regulate cell elongation, and the interaction between the sources of hormones directly or indirectly regulates cell elongation. It has been reported that AtMYB52, AtMYB54, and AtMYB69 regulate the biosynthesis of lignin, xylan, and cellulose, participating in secondary cell wall thickening (Stracke et al. 2001; Zhong et al. 2008). *OsMPH1* may exhibit functions similar to these transcription factors, particularly in cell wall development. In plants, hormones including small organic molecules as well as larger peptides and small proteins act as ligands and interact with receptor proteins to trigger rapid biochemical changes and induce intracellular transcriptional and long-term physiological responses. Receptor kinases have been demonstrated to be important for cell elongation. For example, auxins can stimulate cell elongation by activating ROPs [Rho-like guanosine triphosphatases (GTPase)], and the TMK receptors-like kinases activate ROP activators, such as ROP-GEF, via phosphorylation to activate ROPs [22, 23]. The cell wall-associated receptor kinase WAK4 has been reported to be involved in cell elongation and plant development. WAK4 antisense expression results in cell elongation and developmental arrest [21]. Our further investigation showed that *WAK4* homologous genes in rice, including *LOC*_*Os02g42150*, *LOC*_Os04g30010, *LOC*_*Os09g29560* and *LOC*_*Os04g51030*, were up-regulated in *OsMPH1V* transgenic plants but down-regulated in *OsMPH1E* transgenic plants. A previous study identified 27 AtWAKs in *Arabidopsis* and 130 OsWAKs in rice, suggesting functional diversification in *Arabidopsis* and rice [24, 25]. Therefore, these four genes might be involved in the regulation of cell elongation, differing from the functions observed in *Arabidopsis*. Additional efforts focusing on the functional activity of these genes will be helpful to understand the regulation of cell elongation in rice. Furthermore, we identified a series of indole biosynthetic pathway genes whose expression patterns were changed in the transgenic plants, which implied that auxin-regulated cell elongation is an important component of the variation in plant height.

OsMPH1 comprises an R1-type MYB domain that is presumably required for DNA binding. The MYB proteins are divided into 4 sub-families according to the structure of the DNA binding domain that contains one to three repeats. Each repeat composed of approximately 53 amino acid residues that form a helix-turn-helix. OsMPH1 MYB domain contains 52 amino acid residues and shares highly similarities with reported R1-type MYB proteins. OsMPH1 localizes to the nucleus and possesses weak transcriptional activity which implies that OsMPH1 is typical R1-type MYB protein. Previous research on R1-type MYB genes *OsMYBS1*, *OsMYBS2*, and *OsMYBS3* in rice has demonstrated that these genes display the DNA-binding capacity to bind the TATCCA motif [26]. Thus, we further examined whether a TATCCA motif was present in the promoter regions (1,000 bp upstream) of *OsMPH1*-regulated genes. We found that *LOC*_*Os04g08828*, *LOC*_*Os04g09604*, *LOC*_*Os04g15920*, *LOC*_*Os04g51030* and *LOC*_*Os06g02019* possessed at least one TATCCA motif in their promoter regions, which suggests that these genes might be direct targets of *OsMPH1*.

## Supporting information

S1 Fig Identification of transgenic plants. **A** Immunoblot analysis of WT and *OsMPH1V* plants. **B** *OsMPH1V* and *OsMPH1E* expression level analysis by qRT-PCR. **C** Immunoblot analysis of WT and *OsMPH1-OX* plants. **D** *OsMPH1-OX* expression level analysis by qRT-PCR.
(TIF)

S2 Fig Phenotypic analysis of *OsMPH1* RNAi plants. **A** Gross morphology of WT and *OsMPH1-OX*. Bars = 20 cm. **B** *OsMPH1-OX* expression level analysis by qRT-PCR. **C** Comparison of plant height between WT, *OsMPH1V* and *OsMPH1E* transgenic rice. Data are shown as the means ± s.d. (Student’s *t* tests, **P < 0.01, n = 60).
(TIF)

S3 Fig Analysis of yield traits in *OsMPH1-OX* and *OsMPH1-RNAi* plants. **A-D** Comparison of the number of grains weight per plot, KGW, tillers per plant, grain per panicle between WT, *OsMPH1-OX* and *OsMPH1-RNAi* transgenic rice. Data are shown as the means ± s.d. (Student’s *t* tests, *P < 0.05, **P < 0.01, n = 60).
(TIF)

S4 Fig Heat map of RNA-seq data. (TIF)

S1 Table Primers used in this study. (XLSX)

S2 Table Differentially expressed genes between *OsMPH1V* and *OsMPH1E*. (XLSX)

S3 Table The protein sequences used to build the phylogenetic tree. (XLSX)

## References

[1] Palme K, Li X, Teale WD (2014) Towards second green revolution: engineering nitrogen use efficiency. J Genet Genomics 41: 315–316. doi: 10.1016/j.jgg.2014.05.003 24976120 10.1016/j.jgg.2014.05.003

[2] Mooney BP (2009) The second green revolution? Production of plant-based biodegradable plastics. Biochem J 418: 219–232. doi: 10.1042/BJ20081769 19196243 10.1042/BJ20081769

[3] Wollenweber B, Porter JR, Lubberstedt T (2005) Need for multidisciplinary research towards a second green revolution. Curr Opin Plant Biol 8: 337–341. doi: 10.1016/j.pbi.2005.03.001 15860432 10.1016/j.pbi.2005.03.001

[4] Katiyar A, Smita S, Lenka SK, Rajwanshi R, Chinnusamy V, et al (2012) Genome-wide classification and expression analysis of MYB transcription factor families in rice and Arabidopsis. BMC Genomics 13: 544 doi: 10.1186/1471-2164-13-544 23050870 10.1186/1471-2164-13-544 PMC3542171

[5] Dubos C, Stracke R, Grotewold E, Weisshaar B, Martin C, et al (2010) MYB transcription factors in Arabidopsis. Trends Plant Sci 15: 573–581. doi: 10.1016/j.tplants.2010.06.005 20674465 10.1016/j.tplants.2010.06.005

[6] Feller A, Machemer K, Braun EL, Grotewold E (2011) Evolutionary and comparative analysis of MYB and bHLH plant transcription factors. Plant J 66: 94–116. doi: 10.1111/j.1365-313X.2010.04459.x 21443626 10.1111/j.1365-313X.2010.04459.x

[7] Zhong R, Lee C, Zhou J, McCarthy RL, Ye ZH (2008) A battery of transcription factors involved in the regulation of secondary cell wall biosynthesis in Arabidopsis. Plant Cell 20: 2763–2782. doi: 10.1105/tpc.108.061325 18952777 10.1105/tpc.108.061325 PMC2590737

[8] Ko JH, Kim WC, Han KH (2009) Ectopic expression of MYB46 identifies transcriptional regulatory genes involved in secondary wall biosynthesis in Arabidopsis. Plant J 60: 649–665. doi: 10.1111/j.1365-313X.2009.03989.x 19674407 10.1111/j.1365-313X.2009.03989.x

[9] Cassan-Wang H, Goue N, Saidi MN, Legay S, Sivadon P, et al (2013) Identification of novel transcription factors regulating secondary cell wall formation in Arabidopsis. Front Plant Sci 4: 189 doi: 10.3389/fpls.2013.00189 23781226 10.3389/fpls.2013.00189 PMC3677987

[10] Zhong R, Ye ZH (2012) MYB46 and MYB83 bind to the SMRE sites and directly activate a suite of transcription factors and secondary wall biosynthetic genes. Plant Cell Physiol 53: 368–380. doi: 10.1093/pcp/pcr185 22197883 10.1093/pcp/pcr185

[11] Kim WC, Ko JH, Kim JY, Kim J, Bae HJ, et al (2013) MYB46 directly regulates the gene expression of secondary wall-associated cellulose synthases in Arabidopsis. Plant J 73: 26–36. doi: 10.1111/j.1365-313x.2012.05124.x 26011122 10.1111/j.1365-313x.2012.05124.x

[12] Ko JH, Kim WC, Kim JY, Ahn SJ, Han KH (2012) MYB46-mediated transcriptional regulation of secondary wall biosynthesis. Mol Plant 5: 961–963. doi: 10.1093/mp/sss076 22914575 10.1093/mp/sss076

[13] Zhong R, Richardson EA, Ye ZH (2007) The MYB46 transcription factor is a direct target of SND1 and regulates secondary wall biosynthesis in Arabidopsis. Plant Cell 19: 2776–2792. doi: 10.1105/tpc.107.053678 17890373 10.1105/tpc.107.053678 PMC2048704

[14] Zhong R, Lee C, McCarthy RL, Reeves CK, Jones EG, et al (2011) Transcriptional activation of secondary wall biosynthesis by rice and maize NAC and MYB transcription factors. Plant Cell Physiol 52: 1856–1871. doi: 10.1093/pcp/pcr123 21908441 10.1093/pcp/pcr123

[15] Huang D, Wang S, Zhang B, Shang-Guan K, Shi Y, et al (2015) A Gibberellin-Mediated DELLA-NAC Signaling Cascade Regulates Cellulose Synthesis in Rice. Plant Cell 27: 1681–1696. doi: 10.1105/tpc.15.00015 26002868 10.1105/tpc.15.00015 PMC4498200

[16] Ye Y, Liu B, Zhao M, Wu K, Cheng W, et al (2015) CEF1/OsMYB103L is involved in GA-mediated regulation of secondary wall biosynthesis in rice. Plant Mol Biol 89: 385–401. doi: 10.1007/s11103-015-0376-0 26350403 10.1007/s11103-015-0376-0

[17] Zhao T, Liu J, Li HY, Lin JZ, Bian MD, et al (2015) Using hybrid transcription factors to study gene function in rice. Sci China Life Sci 58: 1160–1162. doi: 10.1007/s11427-015-4937-x 26410798 10.1007/s11427-015-4937-x

[18] Hiei Y, Ohta S, Komari T, Kumashiro T (1994) Effcient transformation of rice (Oryza-sativaL) mediated by agrobacterium and sequence-analysis of the boundaries of the T-DNA. The Plant Journal 6: 271–282. 7920717 10.1046/j.1365-313x.1994.6020271.x

[19] Steffen A, Athanasios T (1994) Transient trasformation of Arabidopsis Leaf protoplasts: a versatile experimental system to study gene expression. The Plant Journal 5: 421–427 8180625 10.1111/j.1365-313x.1994.00421.x

[20] Meng Y, Li H, Wang Q, Liu B, Lin C (2013) Blue Light–Dependent Interaction between Cryptochrome2 and CIB1 Regulates Transcription and Leaf Senescence in Soybean. The Plant Cell Online 25: 4405–4420. 10.1105/tpc.113.116590 PMC3875726 24272488

[21] Lally D, Ingmire P, Tong HY, He ZH (2001) Antisense expression of a cell wall-associated protein kinase, WAK4, inhibits cell elongation and alters morphology. Plant Cell 13: 1317–1331. 11402163 10.1105/tpc.13.6.1317 PMC135583

[22] Xu T, Dai N, Chen J, Nagawa S, Cao M, et al (2014) Cell surface ABP1-TMK auxin-sensing complex activates ROP GTPase signaling. Science 343: 1025–1028. doi: 10.1126/science.1245125 24578577 10.1126/science.1245125 PMC4166562

[23] Xu T, Wen M, Nagawa S, Fu Y, Chen JG, et al (2010) Cell surface- and rho GTPase-based auxin signaling controls cellular interdigitation in Arabidopsis. Cell 143: 99–110. doi: 10.1016/j.cell.2010.09.003 20887895 10.1016/j.cell.2010.09.003 PMC2950838

[24] Verica JA, He ZH (2002) The cell wall-associated kinase (WAK) and WAK-like kinase gene family. Plant Physiol 129: 455–459. doi: 10.1104/pp.011028 12068092 10.1104/pp.011028 PMC1540232

[25] de Oliveira LF, Christoff AP, de Lima JC, de Ross BC, Sachetto-Martins G, et al (2014) The Wall-associated Kinase gene family in rice genomes. Plant Sci 229: 181–192. doi: 10.1016/j.plantsci.2014.09.007 25443845 10.1016/j.plantsci.2014.09.007

[26] Lu CA, Ho TH, Ho SL, Yu SM (2002) Three novel MYB proteins with one DNA binding repeat mediate sugar and hormone regulation of alpha-amylase gene expression. Plant Cell 14: 1963–1980. doi: 10.1105/tpc.001735 12172034 10.1105/tpc.001735 PMC151477

