# Supplementary figures and images for: OsMPH1 regulates plant height and improves grain yield in rice
Source: PLoS One. 2017 Jul 14;12(7):e0180825. doi: 10.1371/journal.pone.0180825 (PMC5510837; doi:10.1371/journal.pone.0180825)

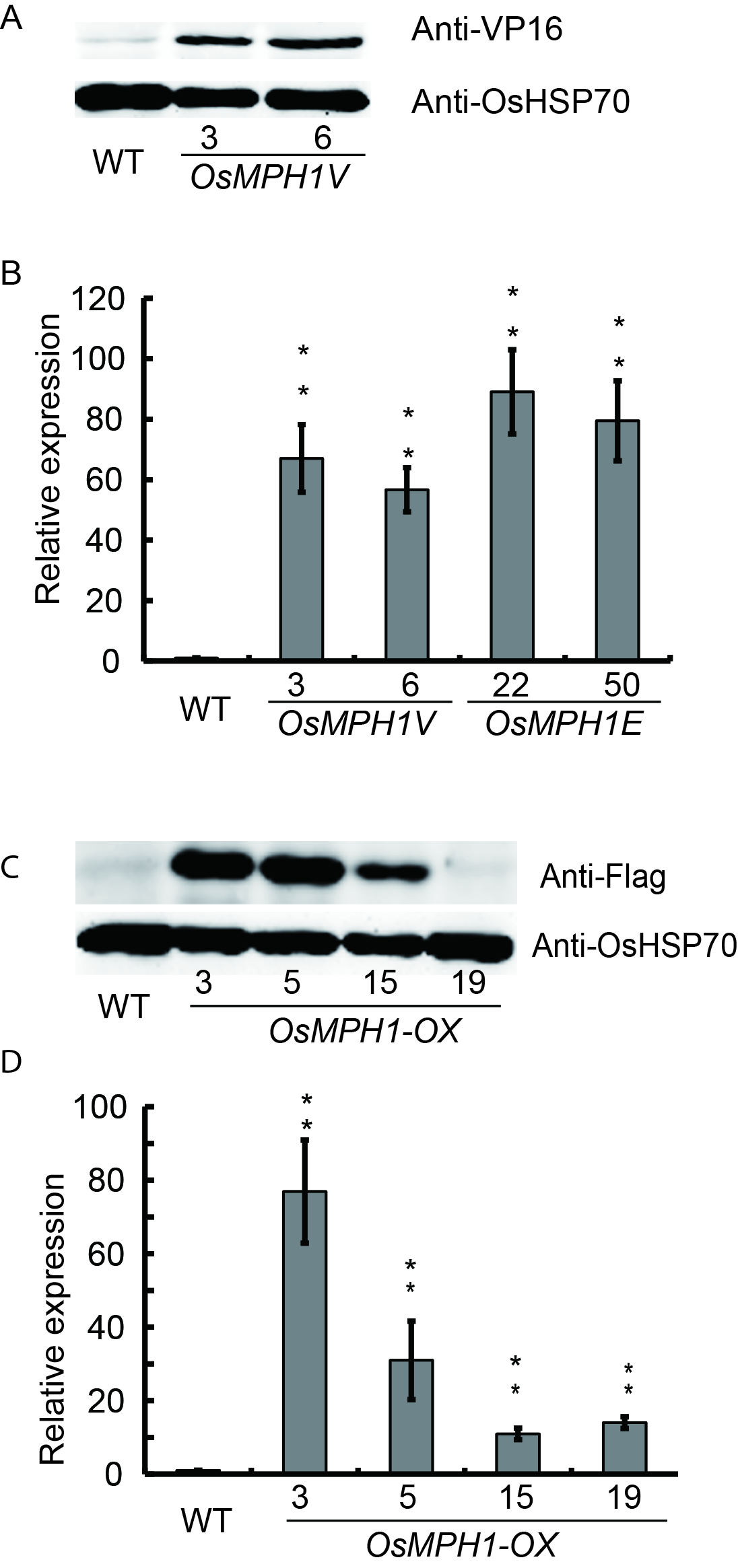

Supplement: S1 Fig — A Immunoblot analysis of WT and OsMPH1V plants. B OsMPH1V and OsMPH1E expression level analysis by qRT-PCR. C Immunoblot analysis of WT and OsMPH1-OX plants. D OsMPH1-OX expression level analysis by qRT-PCR. (TIF) [file pone.0180825.s001.tif]

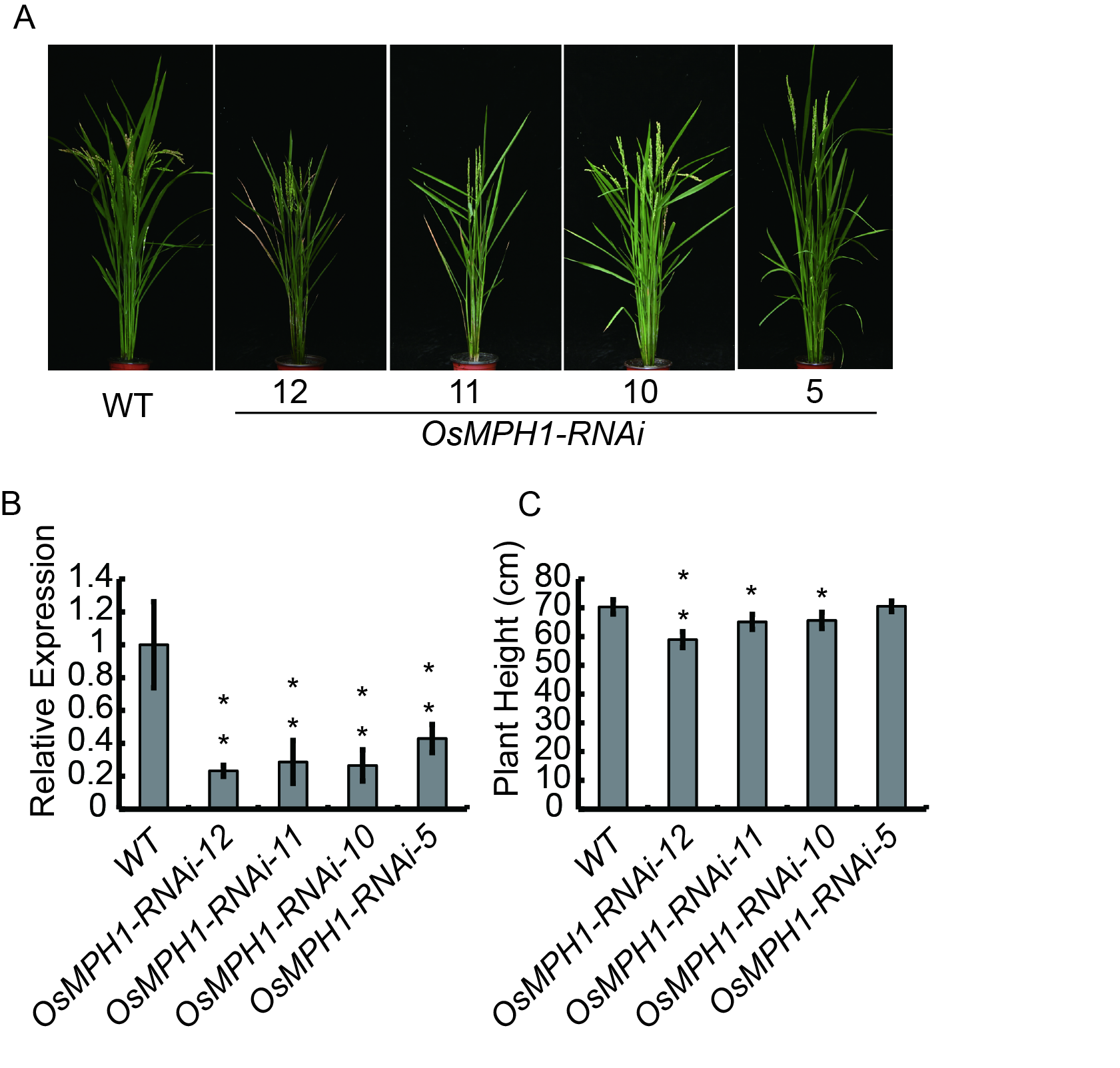

Supplement: S2 Fig — A Gross morphology of WT and OsMPH1-OX. Bars = 20 cm. B OsMPH1-OX expression level analysis by qRT-PCR. C Comparison of plant height between WT, OsMPH1V and OsMPH1E transgenic rice. Data are shown as the means ± s.d. (Student’s t tests, **P < 0.01, n = 60). (TIF) [file pone.0180825.s002.tif]

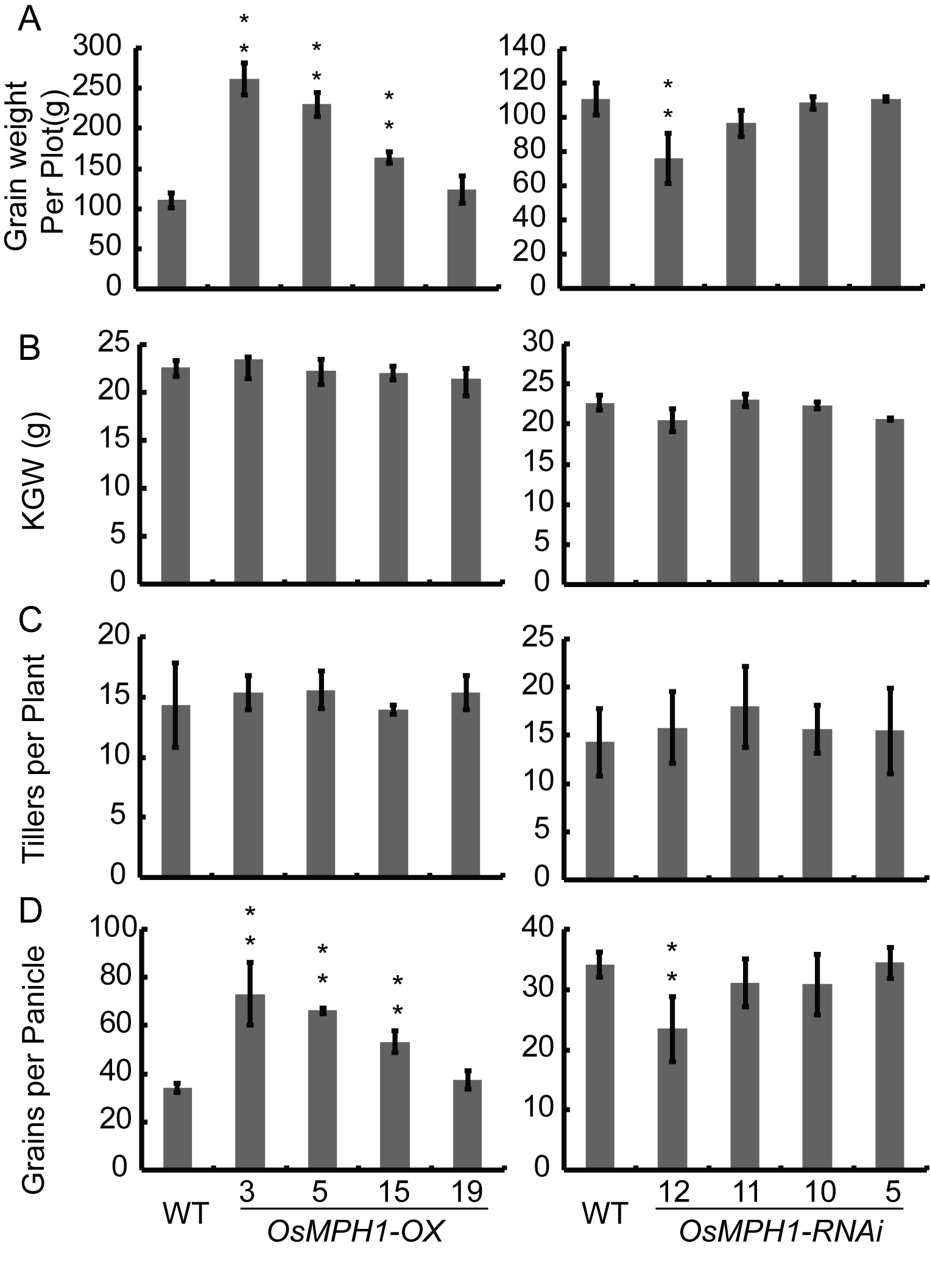

Supplement: S3 Fig — A-D Comparison of the number of grains weight per plot, KGW, tillers per plant, grain per panicle between WT, OsMPH1-OX and OsMPH1-RNAi transgenic rice. Data are shown as the means ± s.d. (Student’s t tests, *P < 0.05, **P < 0.01, n = 60). (TIF) [file pone.0180825.s003.tif]

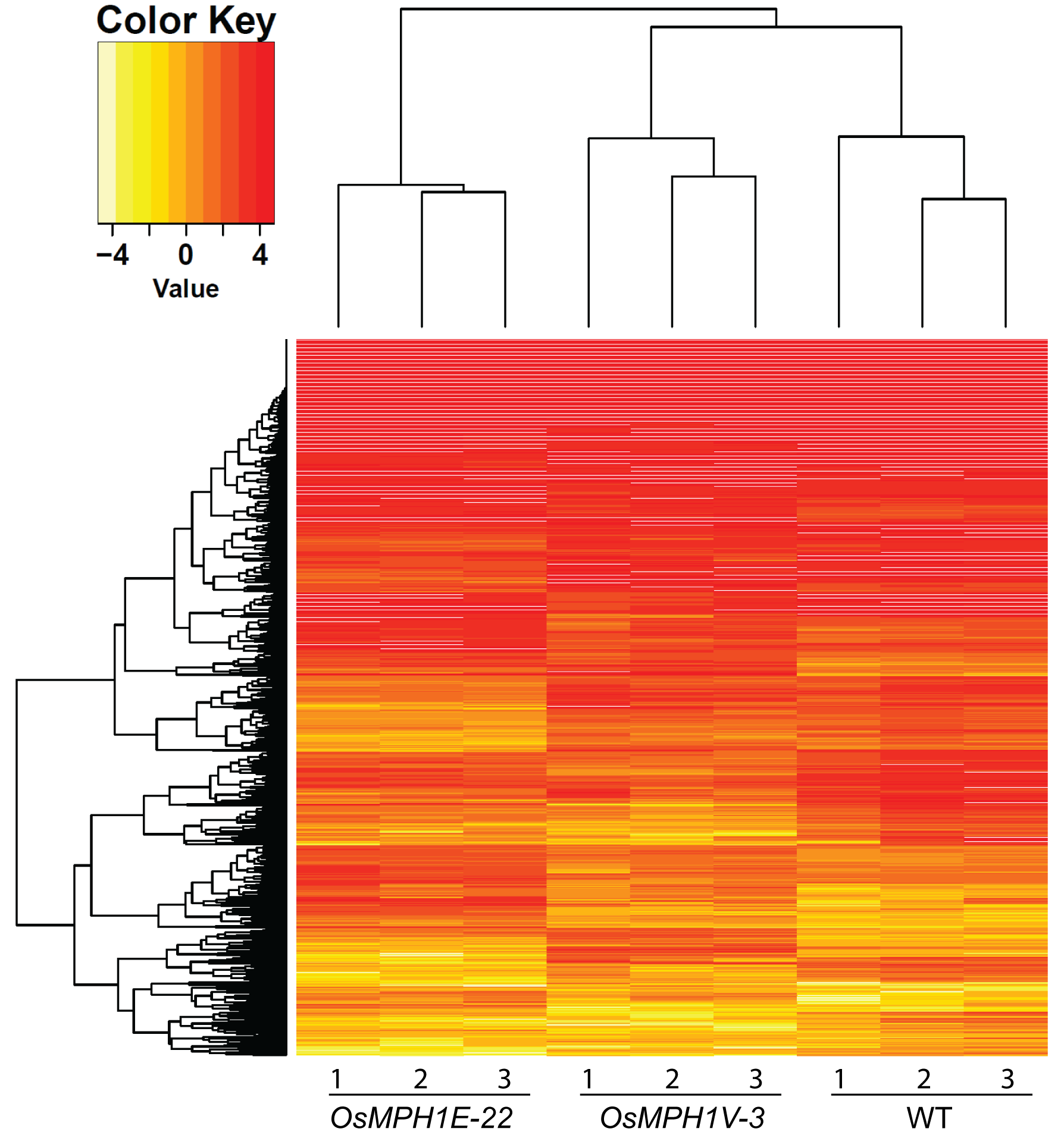

Supplement: S4 Fig — (TIF) [file pone.0180825.s004.tif]
